# Systemic Capillary Leak Syndrome (SCLS) Presentation in Patients Receiving Anti-cancer Treatments

**DOI:** 10.7759/cureus.38335

**Published:** 2023-04-30

**Authors:** Manasa Anipindi, Justyna Kacarow, Daniel Bitetto

**Affiliations:** 1 Internal Medicine, Einstein Medical Center Montgomery, East Norriton, USA; 2 Hospital Medicine, Einstein Medical Center Montgomery, East Norriton, USA

**Keywords:** transplantation, immunotherapy, chemotherapy, cancer care, capillary leak

## Abstract

Systemic capillary leak syndrome (SCLS) is due to increased capillary permeability to proteins and fluid extravasation from blood vessels into surrounding tissues and body cavities. This fluid extravasation leads to hypotension, generalized anasarca, pleural effusions, and pericardial effusions -- the more severe cases of SCLS can cause multiorgan dysfunction, including cardiovascular collapse, shock, and death. The treatment includes corticosteroids, diuretics, albumin, immunoglobulins, and crystalloids. SCLS is potentially fatal. Recognizing signs and symptoms early and treating the patients is essential as this condition is fatal. It sometimes is a diagnosis of exclusion, being very challenging to diagnose and treat. The lack of understanding of the underlying mechanisms causing SCLS and proper treatment guidelines, especially in cancer patients, made diagnosing and treating this condition hard. Reports show that many cancers and anti-cancer treatments, including newer immunotherapy, cause SCLS. The mortality rate of SCLS associated with cytotoxic chemotherapy is 24% at five years. This review focuses on the cancers and anti-cancer drugs causing SCLS, treating acute SCLS, and available preventive regimens. The fundamental purpose of this review is to help clinicians recognize SCLS early to avoid delays in diagnosis and treatment. We also would like to elaborate on the fact that research on cancer-related SCLS is critical for developing staging criteria, useful diagnostic markers, prevention, and treatment strategies for anti-cancer drug-induced SCLS to prevent early discontinuation of anti-cancer drugs.

## Introduction and background

Systemic capillary leak syndrome (SCLS) increases the capillary permeability to proteins, causes endothelial damage, and leads to fluid loss into interstitial space from the intravascular compartment. The classic form is idiopathic, Clarkson's syndrome, or primary SCLS, first described in the 1960s. It constitutes the classic triad of hypoalbuminemia, hemoconcentration, and hypotension [1]. It is fatal, with a mortality rate of 24% at five years. The 5-year and 10-year survival rates were 78% and 69%, respectively, as per European Clarkson disease registry data. Almost 75% of patients have monoclonal gammopathy with idiopathic SCLS [2]. SCLS can be secondary when associated with sepsis, autoimmune diseases, ovarian hyperstimulation syndrome, hemophagocytic lymphohistiocytosis (HLH), viral hemorrhagic fevers, infections, cancers, and chemotherapy drugs [3-6]. SCLS-causing chemotherapy drugs include bendamustine, busulfan, cisplatin, carboplatin, clofarabine, cyclosporine, cyclophosphamide, cytarabine, docetaxel, doxorubicin, daunorubicin, etoposide, fludarabine, gemcitabine, interleukin-2 (IL-2), interleukin-11, melphalan, oxaliplatin, paclitaxel, pemetrexed, treosulfan, thiopeta, and vincristine [7]. Monoclonal antibodies, including rituximab, alemtuzumab, trastuzumab, dinutuximab, bevacizumab, blinatumomab, and nivolumab, can cause SCLS. Drugs like bortezomib, bexarotene, denileukin diftitox, pegaspargase, and pentostatin also cause SCLS. Colony-stimulating factors, including pegfilgrastim, filgrastim, granulocyte monocyte colony-stimulating factor (GM-CSF), granulocyte colony-stimulating factor (G-CSF), and lenograstim, are also associated with SCLS. SCLS is associated with initiating medications like anti‐programmed death checkpoint inhibitors in predisposed patients, like individuals with psoriasis [8]. It is also associated with allogenic bone marrow transplantation [9].

The exact pathogenesis of cancer or drug-related SCLS is unknown. Various studies mention the role of endothelial damage, lymphokine-activated killer cells engagement, cytokines including interleukin-11 (IL-11) and interleukin-12 (IL-12), vascular endothelial growth factor (VEGF), and angiopoietin-2 [10]. Direct toxicity from the drugs leading to endothelial damage is one of the mechanisms of anti-cancer drugs [11]. It is called cytokine release syndrome (CRS) or cytokine storm if it is due to chimeric antigen receptor (CAR) T-cell therapy used in the management of hematologic malignancies [12]. Patients had symptoms within an hour of infusion and correlated with the peak of cytokine levels. Within 24 h, patients with CRS needed to transfer to ICU to manage respiratory distress and for hemodialysis in the setting of acute kidney injury (AKI). Capillary leak syndrome described in patients after allogenic hematopoietic stem cell transplantation is called engraftment syndrome (ES); it leads to pulmonary leak and organ dysfunction. ES is due to the release of inflammatory cytokines and responds to steroids. The symptoms of ES are similar to acute graft versus host disease (GVHD) and further studies are needed to differentiate it from GVHD [13]. Corticosteroids help both acute GVHD and ES; however, not very effective in differentiation syndrome (DS). DS occurs after induction therapy with all-trans-retinoic acid (ATRA) or arsenic trioxide (ATO) in acute promyelocytic leukemia (APL) patients. DS is an adverse side effect noted with isocitrate dehydrogenase (IDH) inhibitors like ivosidenib and enasidenib used in relapsed or refractory IDH-mutated acute myeloid leukemia [14]. The symptoms and signs are like SCLS and include uncontrollable hyperinflammatory reactions in the lungs due to chemokine release causing endothelial damage and transendothelial migration of APL cells [15].

There are no guidelines for the appropriate treatment of secondary causes, and the treatment for secondary SCLS is supportive. We treat the symptoms and signs of SCLS, including pulmonary edema, flu-like symptoms, hypotension, organ failure, and shock [16]. Aggressive fluid administration while following guidelines for sepsis is not the appropriate management for SCLS. That form of treatment can lead to severe anasarca and abdominal compartment syndrome due to volume overload [17-18]. SCLS mimics septic shock, anaphylaxis, and angioedema but can also be confused with polycythemia vera when associated with erythrocytosis [19-21]. The mortality due to SCLS is very high if not diagnosed and treated early. The mortality rate was 15% in patients on prophylactic treatments, whereas the mortality rate was 80% in those not on preventative maintenance treatments [22]. 

The clinical manifestations of SCLS can be due to intravascular volume depletion or extravascular volume overload. The loss of protein-rich fluid into extravascular space activates the renin-angiotensin system causing sodium and water retention. Fluid extravasation into extravascular space causes intravascular volume depletion leading to hypovolemia, AKI, and extravascular volume overload causing pulmonary edema, exudative pleural effusion, pericardial effusion, pulmonary hemorrhage, acute respiratory distress syndrome (ARDS), ascites, abdominal compartment syndrome, muscle edema, intestinal edema, gastric edema, and rarely rhabdomyolysis [23]. AKI is the most common organ injury noted in SCLS due to hypovolemia leading to reduced kidney perfusion [24-25]. Rhabdomyolysis from muscle edema can also lead to renal tubular injury causing AKI [26]. SCLS can also affect the neurological system by increasing vascular permeability in the brain [27-28] (Table 1).

**Table 1:** Presenting signs and symptoms.

Extravascular volume overload:		
Signs:	Peripheral edema, pleural effusion, ascites, pulmonary edema, cerebral edema, and seizures	
Symptoms:	Dyspnea, abdominal distention, paresthesia, and weight gain	
Intravascular volume depletion:		
Signs:	Hypotension, AKI, oliguria, hypoalbuminemia, hemoconcentration, leukocytosis and polycythemia	
Symptoms:	Syncope and generalized weakness. Out of all these hypoalbuminemia is noted in most patients.	
Other findings:		
Signs:		Rhabdomyolysis, leucopenia, thrombocytopenia, immunoglobulin paraprotein

## Review

Systemic capillary leak syndrome and cancers

Several cancers can directly cause SCLS and are associated with 43.6% of cancer-related SCLS. These include B-cell prolymphocytic leukemia, plasma cell leukemia, monoclonal gammopathy, diffuse large B-cell lymphoma, chronic lymphocytic leukemia, multiple myeloma, Sezary syndrome, [29-35] and solid tumors, including lobular carcinoma of the breast, lung, ovarian, rectal, colorectal, pancreatic, and hepatic cancers [36-37]. Per a systematic review of 62 patients, SCLS was most often associated with hematologic malignancy, and about 64.5% were males. Almost 61% of patients had hematologic malignancy, and most had non-Hodgkin lymphoma. The most common presenting symptom in cancer-related SCLS is peripheral edema which was noted in almost 68% of patients, followed by hypotension, pleural effusion, dyspnea, ascites, and others [38]. They are mostly treated with steroids, followed by IV fluids and diuretics. There was no association in patient demographics, clinical, laboratory features, or treatment noticed in patients who passed away with SCLS. SCLS in hematological malignancies had decreased survival [38]. 

Systemic capillary leak syndrome and anti-cancer drugs

Anti-cancer drugs are responsible for 51.6% of cancer-related SCLS, and it is often a lethal complication of cytotoxic chemotherapy, with almost 24% mortality at five years [39]. A retrospective observational study of all the case reports in over 130 countries reported that about 86.5% of SCLS cases from drugs are due to antineoplastic and immunomodulatory drugs. Pulmonary edema is the most common clinical presentation, and this study's SCLS death rate is 27% [40]. The Mertz et al. study states that most SCLS cases occur almost eight days after starting antineoplastic drugs. The highest incidence of SCLS is in patients treated with gemcitabine, clofarabine, and dinileukin diftifox. The common anti-cancer drugs causing SCLS include gemcitabine, clofarabine, fludarabine, cytarabine, pemetrexed, docetaxel, paclitaxel, vincristine, etoposide, oxaliplatin, cyclophosphamide, melphalan, bortezomib, imatinib, and denileukin diftitox [41-42]. Tagraxofusp, a CD123-directed cytotoxin used in blastic plasmacytoid dendritic-cell neoplasm, also causes SCLS [43]. CAR-T cell therapy used in treating hematological cancers is also associated with SCLS due to severe cytokine release [44-45]. Using vincristine in Wilms tumor treatment caused SCLS in the form of pulmonary edema and interstitial pneumonia [46]. High-dose interleukin-2 (IL-2) used in renal cell carcinoma and melanoma is associated with fluid accumulation in extravascular space [47]. Similarly, SCLS occurred in a breast cancer patient receiving capecitabine and trastuzumab, a lymphoma patient receiving rituximab, and a dermatomyositis patient with hepatocellular carcinoma rituximab, and in a colon cancer patient on oxaliplatin. A cutaneous T-cell lymphoma patient receiving IL-2 fusion toxin infusion and a primary hepatic cancer patient receiving interleukin-11 had SCLS [48-54]. Almost 64% of patients treated with IL-2 experienced severe SCLS [55]. Neoadjuvant therapy with gemcitabine and cisplatin in a patient with bladder cancer led to developing edema, hypoproteinemia, and hypotension. Gemcitabine associated with SCLS is reported in many cases [56-57]. Rituximab causes SCLS in almost every patient with a lymphocyte count of >50,000/dL when used to treat chronic lymphocytic leukemia [58]. Docetaxel is also associated with SCLS [59-61]. SCLS is common in patients receiving supportive treatment with recombinant colony-stimulating factors, including filgrastim and pegfilgrastim, but not with others, including tevagrastim, ratiograstim, and biograstim or to-filgrastim [62-64]. SCLS limited the use of anti-tumor drugs in several preclinical studies [65-69]. The incidence of SCLS due to chemotherapy drug regimens ranged from 1% to 100% in meta-analysis and systematic review studies. No specific formal grading criteria exist for SCLS. CRS has five grades based on the severity of illness, and treatment of CRS depends on these grades. 

Treatment 

The management of idiopathic SCLS depends on the hemodynamic stability of the patient. In the severe capillary leak phase with hypotension, shock, and AKI, volume resuscitation with crystalloids remains the primary treatment. If the shock persists after IV fluid administration, using vasopressors, albumin, and hetastarch is considered. Diuretics are helpful in the recovery phase or for patients with the mild capillary leak. In patients with marginal blood pressure with volume overload and preserved perfusion, the combination of albumin and loop diuretics is helpful for fluid removal. Hemodialysis is beneficial in patients with AKI and oliguria. Continuous renal replacement therapy is helpful in patients with hemodynamic instability and those with marked anasarca. Treatment with colloid replacement and paracentesis improves respiratory distress in patients with significant ascites and hypovolemia. The treatment of cancer-related SCLS can differ from idiopathic SCLS. 

Systemic Capillary Leak Syndrome and Chemotherapy

In a 65-year-old female with relapsed multiple myeloma, albumin and IV diuretics did not prevent bortezomib-induced SCLS, and the patient required dialysis. Administration of dexamethasone 20 mg daily with bortezomib 1 mg/m2 on days 1, 4, 8, and 11 avoided the development of the third episode of SCLS [70]. A patient with stage II pancreatic head adenocarcinoma who developed gemcitabine-induced SCLS methylprednisolone at 1 mg/kg for one week improved the symptoms. The patient was discharged with oral prednisone 10 mg daily, preventing further attacks [71]. A dose of 40 mg methylprednisolone and a high amount of furosemide 250 mg helped another patient with pancreatic cancer who developed gemcitabine-induced SCLS [72]. A 54-year-old male with a history of urothelial cancer who received neoadjuvant chemotherapy with cisplatin and gemcitabine required prednisone at 50 mg daily with diuresis for recovery [73]. Methylprednisolone, 1 mg/kg for two weeks, also helped a 56-year-old pancreatic adenocarcinoma who received gemcitabine and a 48-year-old female with stage II breast cancer who received doxorubicin, cyclophosphamide for four weeks followed by paclitaxel weekly for 12 weeks [74]. Chemokine receptor antagonists, such as the use of C-X-C motif chemokine receptor 1 (CXCR1)/CXC chemokine receptor-2 (CXCR2) antagonists or C-C motif chemokine receptor 2 (CCR2)/CXC chemokine receptor-2 (CXCR2) antagonists, are suggested for the treatment in acute promyelocytic leukemia patients with DS who received ATRA or ATO considering the involvement of chemokines and their receptors in the pathogenesis of DS [75]. 

Systemic Capillary Leak Syndrome and Immunotherapy

Newer immune checkpoint inhibitors can also cause SCLS due to endothelial damage and cytokine activation associated with their administration [76-77]. Nivolumab is known to cause late-onset SCLS. A month after completing treatment with nivolumab led to the development of SCLS in a 51-year-old melanoma patient. Even though three days of IV steroids at a dose of 2 mg/kg did not have a significant effect, administering intravenous immunoglobulins (IVIG) at 2 g/kg for four days resolved the symptoms. Hence, the authors suggested using IVIG as the first-line treatment in patients who present with SCLS after discontinuing nivolumab therapy. Authors also noted that continuing IVIG for three more cycles at 400 mg/kg dose every four weeks prevented recurrent attacks [78]. A 43-year woman with stage IIB melanoma treated with nivolumab had a similar presentation three months after finishing the treatment, and IVIG administration improved the chylothorax. This patient received 0.4 g/kg of IVIG for five days, followed by 1 g/kg/day for two consecutive days monthly for five months, and a year later, the follow-up did not show any recurrence [79]. Another 54-year-old patient with malignant nodular melanoma developed pembrolizumab-induced SCLS a month after completing treatment. High-dose corticosteroids, tocilizumab, IV diuretics, and albumin did not help. However, a regimen with IVIG at 0.4 g/kg/day for five days and a tyrosine kinase inhibitor, axitinib at 5 mg twice daily, improved the symptoms. He continued the treatment with 0.4/kg IVIG weekly for 10 weeks and axitinib twice daily for his cancer treatment with notable improvement and reasonable control of SCLS [80]. Ipilimumab is also associated with developing life-threatening SCLS [81-82]. 

Diagnostic markers

The diagnostic triad of idiopathic SLCS includes hypoalbuminemia, with an albumin level of less than 3 g/dL, hemoconcentration with a hematocrit of more than 49%-50% in men, and a hematocrit of 43%-45% in women or hemoglobin of more than 20 g/dL and hypotension with a systolic blood pressure of <90 mmHg. Central venous pressure is as low as 2 mmHg in acute SCLS leak phase patients. Idiopathic SCLS showed increased counts of circulating CD25+ T cells and CD8+ T cell perivascular infiltration. Sera of patients with acute SCLS showed elevation of seven cytokines, CXCL10 (C-X-C motif chemokine ligand 10), angiopoietin-2 chemokine ligand 2 (CCL2), interleukin-12, interleukin-1 beta, interleukin-6, interleukin-8, and tumor necrosis factor-alpha. The sera of both basal and acute SCLS patients showed consistent elevation of CXCL10 compared to controls. Still, its use as a diagnostic marker is limited as other diseases like rheumatoid arthritis, systemic lupus erythematosus, and systemic sclerosis also show elevated levels of CXCL10. Elevation of plasma VEGF, a potential mediator of endothelial dysfunction, is also noted in acute SCLS flare.

Of these cytokines, VEGF elevation indicates the onset of acute SCLS flare and correlates with the episode's severity. About 68% of patients with idiopathic SCLS had IgG kappa isotope of monoclonal gammopathy of undetermined significance (MGUS), but the diagnosis of MGUS is unnecessary for SCLS diagnosis. Complement levels, including C3, C4, and CH50, are low in patients with SCLS due to the leakage of complement proteins into extravascular space. In all suspected SCLS patients, physicians should check the complement factor 1 esterase inhibitor levels to rule out hereditary or acquired angioedema. In patients with monoclonal antibodies induced SCLS, an increase in multiple cytokines such as tumor necrosis factor-α (TNF-α), interferon-gamma, interleukin-2, interleukin-4, and interleukin-6 was noted. ES that occurs after hematopoietic stem cell transplantation is similar to SCLS. It is associated with the release of interleukin-1, TNF-α, and interferon-gamma.

High levels of interleukin-1 beta (β), interleukin-8, and interleukin-12 are noted in acute SCLS patients sera compared to controls [83]. No official diagnostic markers are currently available for cancer-related SCLS and early recognition of SCLS unless the patient develops symptoms. The pathogenic mechanisms involved in cancer and anti-cancer treatments are mainly unknown. Systematic reviews discussed the role of cytokines, angiopoietin-2, chemokines, and VEGF. Gemcitabine causes direct toxicity to the cells leading to increased vascular permeability. The active metabolites of gemcitabine injure mitochondria and induce massive production of reactive oxygen species disrupting the capillary endothelial barrier. A decrease in albumin in initial cancer treatment days has suggested a predictor of capillary leak syndrome when treated with gemcitabine. In hematological malignancies, the systemic release of cytokines leads to vascular leak. An increase or change in C-reactive protein levels might help identify patients at risk for severe CRS. Patients with IL-2-related SCLS had increased levels of angiopoietin two with peak levels on day three after the infusion [84].

Secondary prophylaxis

Theophylline is a well-known prophylactic drug in idiopathic SCLS. A 48-year-old male patient with a history of monoclonal gammopathy had recurrent attacks of idiopathic SLCS, a preventative treatment with theophylline and terbutaline helped. Theophylline blood level must be more than 15 mg/dL to avoid attacks. In a study that evaluated 35 patients with confirmed SCLS, about 79% (23/29) of patients experienced breakthrough episodes while on the regimen of terbutaline and theophylline, and the majority of the patients (20/22) were episode free with the administration of monthly IVIG infusions at a dose of 2 g/kg. In another six-year-old patient, treatment with terbutaline to achieve a serum level of 20 mg/dL, methylprednisolone, and IVIG, antibiotics did not help. Still, as the serum tumor necrosis factor was high, initiating treatment with infliximab at 10 mg/kg helped. This patient's treatment with prophylactic terbutaline and theophylline prevented the SCLS attacks. TNF antagonists are helpful if TNF-α is elevated [85]. As noted earlier, administering steroids with anti-cancer drugs (bortezomib, gemcitabine) avoided the recurrence of SCLS. Based on the literature review, docetaxel-induced SCLS in breast cancer patients is decreased by giving methylprednisolone 40 mg 24 h before and continuing it for 48 h after docetaxel administration [86]. Administration of IVIG weekly for 4-10 weeks prevented SCLS relapse in patients receiving immunotherapy. Case reports and literature support using corticosteroids and diuretics to reduce the effects of capillary leak syndrome induced by chemotherapy [87-88]. Bevacizumab, an anti-VEGF antibody, has also been reported in treating SCLS associated with immunotherapy. Tocilizumab and dexamethasone can reduce the risk of CRS and AKI in patients receiving CAR T cell therapy [89-91]. Some patients required dialysis and fluid management, including paracentesis and thoracentesis. These prophylactic or treatment strategies are from observational studies, not randomized control clinical trials. 

Steroids work by decreasing cytokine expression; steroids also have a role in avoiding cytokine-mediated endothelial damage but are not efficient if the mechanism of the capillary leak is due to endothelium-damaging substances. Diuretics work by removing extra fluid from accumulating and amiloride is preferred as it has a lesser blood pressure-lowering effect [92]. Immunoglobulins can reduce capillary leaks, prevent relapses, and improve SCLS survival by preventing complement from attacking cells [93-94]. Methylxanthines decrease the effects of cytokines or other mediators that cause endothelial damage and vascular hyperpermeability. Beta-agonists like terbutaline decrease macromolecular leakage by inhibiting the effects of histamine and bradykinin. Both theophylline and terbutaline increase intracellular cyclic adenosine monophosphate, which inhibits capillary permeability [95-96]. The use of terbutaline and steroids also have a role in reducing the frequency of SCLS attacks. 

## Conclusions

Many case reports and review articles are available online showing the effect of SCLS with chemotherapy and the percentage of patients affected. Based on systematic review studies, the incidence of SCLS is 100% in patients receiving interleukin-2 and bevacizumab, and the incidence of SCLS in patients on gemcitabine is only 3.5%. But we need to find out the exact number of patients who have had SCLS as a side effect of cancers and the anti-cancer regimes, as some of them can go unreported. Given the limited research and clinical experience with this syndrome, it is also essential to maintain a national or international registry to provide a further understanding of the clinical symptomatology and management plan. 

The prognosis of SCLS is poor, as per studies. So close monitoring for SCLS symptoms is critical, especially in patients on anti-cancer treatments. Diagnosing this condition early enough is essential to avoid significant morbidity and mortality. It is also important to realize that it mimics so many other diseases, and timely diagnosis can sometimes be difficult with delays in treatment. There are secondary prophylactic treatments to avoid the recurrence of SCLS, but the literature review did not reveal any primary preventive medications that can prevent SCLS altogether. Identifying markers and developing inhibitors against them might prevent SCLS. For example, developing drugs like angiopoietin-2 inhibitors for patients receiving high-dose interleukin-2 therapy can avoid SCLS, a significant dose-limiting side effect, thereby preventing early withdrawal of IL-2 therapy. The inability to continue cancer treatment due to SCLS can be a limiting factor, so it is essential to develop diagnostic markers, primary prevention, and definitive therapies for anti-cancer treatment-related SCLS. It is imperative also to treat life-threatening SCLS during anti-cancer therapies while maintaining the anti-tumor effect, so it is crucial to design grading systems and treatment algorithms for patients at risk for SCLS on anti-cancer treatment regimens to avoid early anti-cancer drug dosage reductions and treatment withdrawal. 
